# Functional changes in the salience network of patients with amnesic mild cognitive impairment before and after repetitive transcranial magnetic stimulation

**DOI:** 10.1002/brb3.3169

**Published:** 2023-08-03

**Authors:** Qianqian Yuan, Chen Xue, Xuhong Liang, Wenzhang Qi, Shanshan chen, Yu Song, Huimin Wu, Xulian Zhang, Chaoyong Xiao, Jiu Chen

**Affiliations:** ^1^ Department of Radiology The Affiliated Brain Hospital of Nanjing Medical University Nanjing China; ^2^ Department of Radiology The Affiliated Drum Tower Hospital, Medical School of Nanjing University Nanjing China; ^3^ Institute of Medical Imaging and Artificial Intelligence Nanjing University Nanjing China; ^4^ Medical Imaging Center The Affiliated Drum Tower Hospital, Medical School of Nanjing University Nanjing China

**Keywords:** Alzheimer's disease (AD), amnesic mild cognitive impairment (aMCI), functional connectivity (FC), precuneus (PCUN), repetitive transcranial magnetic stimulation (rTMS)

## Abstract

**Background:**

Amnestic mild cognitive impairment (aMCI) is considered to be the prodromal stage of Alzheimer's disease (AD). The precuneus (PCUN) may be an imaging marker for monitoring the progression of AD. Meanwhile, cognitive impairment in AD patients is closely related to functional connectivity (FC) changes in the salience network (SN). We hypothesize that there are specific neuroimaging biomarkers in the SN and that FC changes in aMCI patients after repetitive transcranial magnetic stimulation (rTMS) intervention are associated with cognitive function. The purpose of this study was to first investigate the pattern of functional changes in aMCI patients and healthy controls (HCs) and then compare the functional changes in aMCI patients before and after rTMS targeting to PCUN and its correlation with cognitive function.

**Methods:**

Thirty‐six HCs and 61 aMCIs were recruited for our study. Eleven people in the aMCI group received rTMS intervention 5 days a week for 4 weeks. Using the right anterior insula as the seed‐of‐interest, we first compared FC changes in HC and aMCI patients and then compared cognitive function in aMCI patients before and after rTMS. The above is the functional connection analysis of seed‐to‐voxel. Moreover, we investigated the FC changes in aMCI patients after rTMS intervention and its correlation with cognitive function.

**Results:**

Compared with HC, the aMCI group showed altered FC in bilateral parahippocampal gyrus, bilateral inferior parietal lobule, left middle frontal gyrus, and left middle temporal gyrus. Moreover, rTMS at PCUN improved the cognitive function of aMCI patients, which was related to the altered FC in posterior cerebellar lobes (CPL).

**Conclusions:**

Our findings suggest that rTMS targeting PCUN is a promising, noninvasive approach to ameliorating cognitive dysfunction in aMCI patients, and that this cognitive improvement is accompanied by brain connectivity modulation.

## Graphical abstract

Our study used the right anterior insula as the seed point for functional connectivity (FC) calculation of the two groups of subjects. First, we compared the FC changes between the healthy control (HC) and amnestic mild cognitive impairment (aMCI) groups. Second, changes in the cognitive function of aMCI patients before and after repeated transcranial magnetic stimulation (TMS) were compared. Finally, we compared the FC changes before and after repeated TMS and analyzed the correlation between the different brain regions and cognitive function.

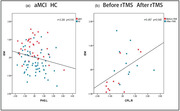

## 1 INTRODUCTION

Alzheimer's disease (AD), the most common cause of dementia, is a growing global health problem with huge impacts on individuals and societies (Lane et al., 2018). Amnestic mild cognitive impairment (aMCI), characterized by memory decline, is considered an intermediate stage between healthy aging and dementia, with a high risk of developing Alzheimer's dementia (Xue et al., 2019; Yuan et al., 2021). Currently, there are no effective treatments to change the underlying pathology and disease course of AD (Lane et al., 2018). At the same time, some studies highlight the urgent need for new treatments to improve cognition (Xue et al., 2019; Yao et al., 2022).

In recent years, repetitive transcranial magnetic stimulation (rTMS) has been increasingly used as a noninvasive means to modulate cortical physiology, which is a promising treatment for aMCI and AD (Beynel et al., 2020; Chou et al., 2020). rTMS is a noninvasive neuromodulation technique that uses stimulation coils to deliver electromagnetic pulses that induce electrical currents in the brain to regulate neural tissue (Beynel et al., 2020). The exact mechanism of TMS is unclear, but studies have reported that TMS can act on the stimulation site, as well as distal sites associated with the proximal stimulation site, to regulate specific neural network activity (Beynel et al., 2020; Yao et al., 2022). In general, stimulation frequencies of 5–20 Hz generally increase cortical responses and are associated with behavioral facilitation (Guse et al., 2010; Speer et al., 2000). In contrast, stimulation at frequencies below 5 Hz tends to result in decreased cerebral blood flow and inhibition of neural responses and behavior (Chen et al., 1997; Speer et al., 2000). In contrast to rTMS, repetitive rTMS can produce long‐lasting effects on neural activity and behavior after the stimulation period (Chou et al., 2015; Fitzgerald et al., 2006). Moreover, because the brain can be viewed as an interacting regional connectome, rTMS‐induced changes are not limited to the stimulated functional network but also spread to other brain networks (Bassett & Sporns, 2017; Bohning et al., 1998; Navarro De Lara et al., 2015). In our study, the target of rTMS stimulation was precuneus (PCUN). PCUN is an important component of default mode network (DMN) and has been highlighted as a key region for memory impairment observed in early AD (Koch et al., 2018). It is worth noting that the PCUN is also the most prominent area of tau protein pathological deposition and neuroinflammation (Koch et al., 2022; Veitch et al., 2019). At the same time, previous studies have reported that the use of high‐frequency rTMS in the treatment of AD has achieved a very good effect, effectively improving the memory function of AD patients (Chen et al., 2020; Koch et al., 2018). Therefore, it is reasonable to speculate that PCUN as a target for rTMS stimulation may be beneficial to memory improvement in aMCI patients to some extent.

Some studies have shown that resting‐state functional magnetic resonance imaging (fMRI) has become an important tool in cognitive research (Sporns et al., 2005; Wang et al., 2013). In this study, we evaluated the association between neural activity in different regions of the brain space by correlation of blood oxygen level correlation time series (Balachandar et al., 2015; Yuan et al., 2021). To our knowledge, salience network (SN) consists mainly of the anterior cingulate cortex and anterior insula, which are involved in emotional processes, attention, and interoception (Sridharan et al., 2008; Xue et al., 2021); SN plays a central role in the triple network model of DMN, SN, and executive control network (ECN) proposed by Menon in 2011. When a significant event is detected, SN can activate brain networks that direct the DMN and ECN to perform cognitive tasks and help the corresponding brain regions respond appropriately to stimuli (Uddin, 2015; Xue et al., 2021). There are few and inconsistent studies on SN network changes in the AD disease spectrum. Multiple studies have shown that patients with AD have increased SN functional connectivity (FC) compared to normal controls (Menon & Uddin, 2010; Uddin, 2015; Yu et al., 2019). Meanwhile, previous studies have shown that SN plays a crucial role in higher cognitive function and is closely related to FC changes in patients with aMCI (Xue et al., 2021). Therefore, further study of SN changes in aMCI will help us better understand its pathological mechanism and provide a theoretical basis for clinical search for ways to delay the progression of AD (Sporns et al., 2005).

It should be noted that in this study, all FC changes were calculated with right anterior insula (rAI) as the seed point. First, we compared aMCI and healthy control (HC) to look for the brain regions where FC is altered. At the same time, we speculate that these different brain regions can be effective biomarkers of AD conversion. In addition, we hypothesized that a 4‐week intervention using rsfMRI studies targeting PCUN as an rTMS stimulus could improve cognition and regulate specific brain regions in patients with aMCI. Specifically, the combination of rTMS and fMRI may help to understand the role of PCUN in the cognitive regulation of AD and further provide new ideas for the search for effective interventions in AD.

## 2 MATERIALS AND METHODS

### 2.1 Subjects

The data for the applied research were obtained from our in‐home database: Nanjing Brain Hospital‐Alzheimer's Disease Spectrum Neuroimaging Project 2 (NBH‐ADsnp2) (Nanjing, China), which is continuously being updated. Information on NBH‐ADsnp2 is provided in Section S1. Volunteers were recruited from hospitals and local communities through advertising and radio, and a total of 286 data were collected for the current study. We selected 86 aMCI patients and 38 HCs for the study. After strict exclusion criteria, 61 aMCI patients and 36 HC patients were finally included in the study, with a total of 99 cases of data. Eleven of the 61 patients with aMCI underwent subsequent rTMS intervention and data reacquisition. It should be noted that only a small number of subjects received sham stimulation, and we did not study the sham stimulation group. Inclusion and exclusion criteria for participants are provided in Section S1.

### 2.2 Ethical principle

This study was approved by the Human Participants Ethics Committee of the Affiliated Brain Hospital of Nanjing Medical University (No. 2018‐KY010‐01 and No. 2020‐KY010‐02). Written informed consents were obtained from all subjects.

### 2.3 Neurocognitive assessments

All subjects underwent comprehensive and standardized clinical assessment interviews, including demographic questionnaires, medical history, neurological and mental status examinations, and MRI scans. We also performed a comprehensive and standard neurocognitive assessment of episodic memory (EM), executive function (EF), visuospatial function, and information processing speed for all participants (Chen et al., 2020; Xue et al., 2021). The individual raw score of each neuropsychological test was transformed to normalized *Z* scores. For a scale in time, we take the inverse of its *Z* score. Subsequently, the normalized *Z* score was averaged to calculate the composite *Z* score of each cognitive domain. (Refer to Section S2 for details of the assessment.) All assessments were performed by two experienced clinicians.

### 2.4 MRI data acquisition

The acquired images include resting‐state fMRI images and structural MRI images. We first completed the neurocognitive scale assessment, followed by MRI scans, and then started the 4‐week rTMS intervention. After 4 weeks of intervention, MRI scans were performed again to collect image data. The details regarding image acquisition parameters are provided in Section S3.

### 2.5 Preprocessing of rsfMRI Data

Consistent with previous studies, all fMRI images were implemented in MATLAB2013b using the DPABI‐based SPM program (Chen et al., 2016, 2020). First, after discarding the first 10 volumes, the remaining voxels were corrected for slice time and head motion. The structural images were then segmented into gray matter, white matter, and cerebrospinal fluid using the DARTEL technique, and gray matter was used as a covariate in subsequent statistical analyses (Ashburner & Friston, 2009). Then, the 24 motor parameters, global signal, white matter signal, and CSF signal were subjected to segmentation and interference covariate regression. The filtering frequency was chosen to be 0.01–0.08 Hz (Li et al., 2018), and the segmented T1 image was used for normalization and resampling to an isotropic voxel size of 3 mm. Finally, all fMRI images were smoothed using a 6 × 6 × 6 mm^3^ FWHM Gaussian kernel. After the preprocessing is completed, we conduct statistical analysis on the preprocessed data.

### 2.6 Functional connectivity analysis

Resting‐state FC analysis was performed to show direct functional coupling between brain regions. According to previous studies, the rAI formed a region of interest with a radius of 6 mm (MNI space: 38, 22, and −10), which we defined as ROI (Wotruba et al., 2014; Xue et al., 2019). First, the average time series of ROI were extracted, and then the average time series in the ROI and the whole brain in the GM mask (obtained by preprocessing) were analyzed by voxel‐level correlation. Finally, Fisher's *Z* transform was used to enhance the normality of the correlation coefficients (Uddin, 2015).

### 2.7 rTMS protocol

The stimulation point of rTMS was the PCUN brain region, which was placed in Pz according to the international 10–20 brain electrode distribution system. The frequency was 10 Hz, and the intensity was 80%–120% of the resting‐state motor threshold (MT) (depending on the specific brain region stimulated by the rTMS). In the contralateral (right) hand relaxed first dorsal interosseous muscle, the motor evoked potential that produced the lowest intensity at least 5 times out of 10 trials was defined as MT60. The coil was placed approximately in the central sulcus and stimulated in the left motor cortical area (M1).

Eleven aMCI subjects were treated 5 times a week for 4 weeks. Specific stimulus parameters are as follows: The continuous stimulation duration was 4 s, the number of stimuli was 40, the interval time was 56 s, the repetitive stimulation was 25 times, 25 min/time, and the total number of pulses was 1000. After TMS treatment, 11 aMCI subjects underwent neurocognitive scale assessment and MRI data collection.

### 2.8 Statistical analyses

Statistical Package for Social Sciences (SPSS) software, version 22.0 (IBM), was used to analyze the demographic and clinical information. The chi‐squared tests and two‐sample *t*‐test were conducted to compare the demographic and neurocognitive data across the two groups with aMCI and HC. Gaussian random field (GRF) correction is used for post hoc verification.

Because our subjects were compared between two groups, we directly conducted two‐sample *t*‐test. The two‐sample *t*‐test was used with the mask resulting from preprocessing. We first compared the functional changes between the HC and aMCI groups and then compared the functional changes before and after rTMS with the paired *t*‐test. To rigorously correct the results, we used GRF corrections were applied with a threshold of *p* < .05 and a cluster size >50 voxels. The FCs of significantly altered regions were extracted with a Resting‐State fMRI Data Analysis Toolkit (REST)^1^ and were later used for correlation analyses. After controlling for the influence of age, sex, education level, and GM volume, we conducted correlation analysis to reveal the relationship between FCs change and cognitive domain in aMCI and the relationship between FCs change and cognitive domain before and after rTMS (*p* < .05).

## 3 RESULTS

### 3.1 Demographic and neurocognitive characteristics

**FIGURE 1:**
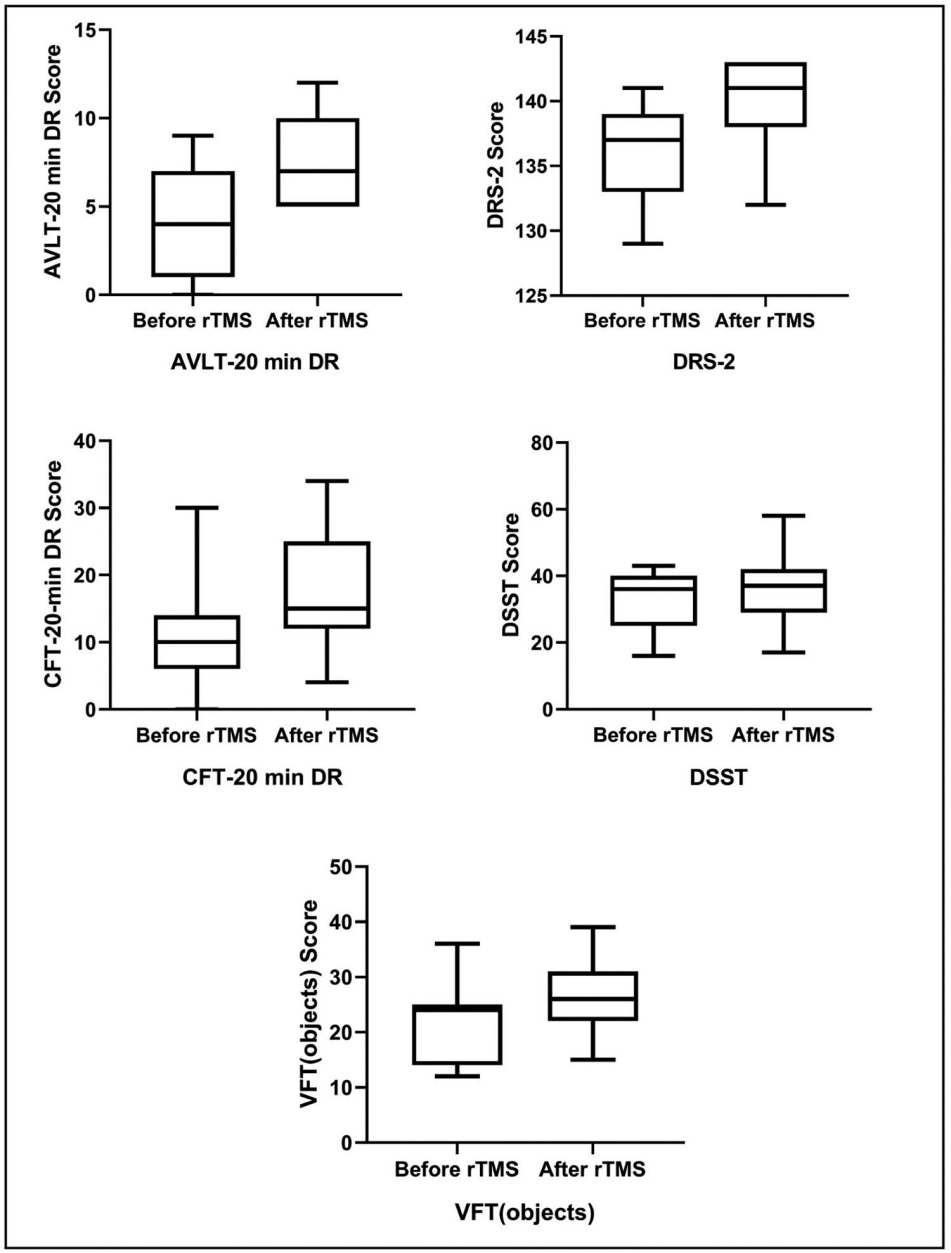
**Mean cognitive performance in before repetitive transcranial magnetic stimulation (rTMS) and after rTMS**. AVLT‐20 min DR, auditory‐verbal learning test delay of 20 min; DRS‐2, dementia rating scale; CFT, complex figure test; DSST, digit symbol substitution test; VFT, verbal fluency test.

The demographic and neurocognitive information of all participants included 36 HCs (mean age 61.78 ± 6.711), 61 aMCI (mean age 65.49 ± 5.833), and 11 aMCI patients who underwent 2 courses of rTMS (Table 1). As expected, the results of the study showed that DRS‐2, AVLT‐20‐min DR, CFT‐20‐min DR, digit symbol substitution test, and verbal fluency test (objects) scores were all higher in aMCI patients after rTMS compared to those before rTMS (*p* < .05, Figure 1).

**TABLE 1:** Demographic and clinical characteristics of the participants.

Characteristics	HC	aMCI	*t*‐Values (*χ* ^2^)	*p*‐Values	Before rTMS	After rTMS	*t*‐Values (*χ* ^2^)	*p*‐Values
	*n* = 36	*n* = 61			*n* = 11	*n* = 11		
Age (years)	61.78 (6.711)	65.49 (5.833)	.014	.906	65.82 (7.534)	66.45 (7.647)	.027	.872
Gender (male/female)	15/21	21/40	1.632	.204	2/9	2/9	0.000	1.000
Education level (years)	11.82 (2.256)	11.44 (2.811)	2.432	.122	12.45 (3.228)	12.45 (3.228)	.008	.929
**Composite *Z* scores of each cognitive domain**								
Episodic memory	0.51 (0.57)	−0.29 (0.60)	.001	.97	0.33 (0.66)	−0.33 (0.71)	.149	.704
Information processing speed	−1.19 (2.57)	0.24 (6.07)	1.456	.231	−3.90 (7.13)	−1.16 (1.75)	5.512	.029^a^
Executive function	0.20 (0.77)	−0.11 (0.79)	.44	.509	0.18 (0.68)	−0.18 (0.97)	1.947	.178
Visuospatial function	0.15 (1.31)	−0.19 (1.15)	.356	.552	−1.27 (3.78)	−0.29 (0.63)	2.833	.108

*Note*: Data are presented as mean (standard deviation, SD).

Abbreviations: aMCI, amnestic mild cognitive impairment; HC, healthy controls; rTMS, repetitive transcranial magnetic stimulation.

^a^ Significant differences were found among before and after aMCI subjects. Most *p*‐values were obtained using two‐sample *t*‐test, except for gender (chi‐square test). This study utilized the composite *Z* scores to determine the level of each cognitive domain.

### 3.2 Functional connectivity of rAI

**FIGURE 2:**
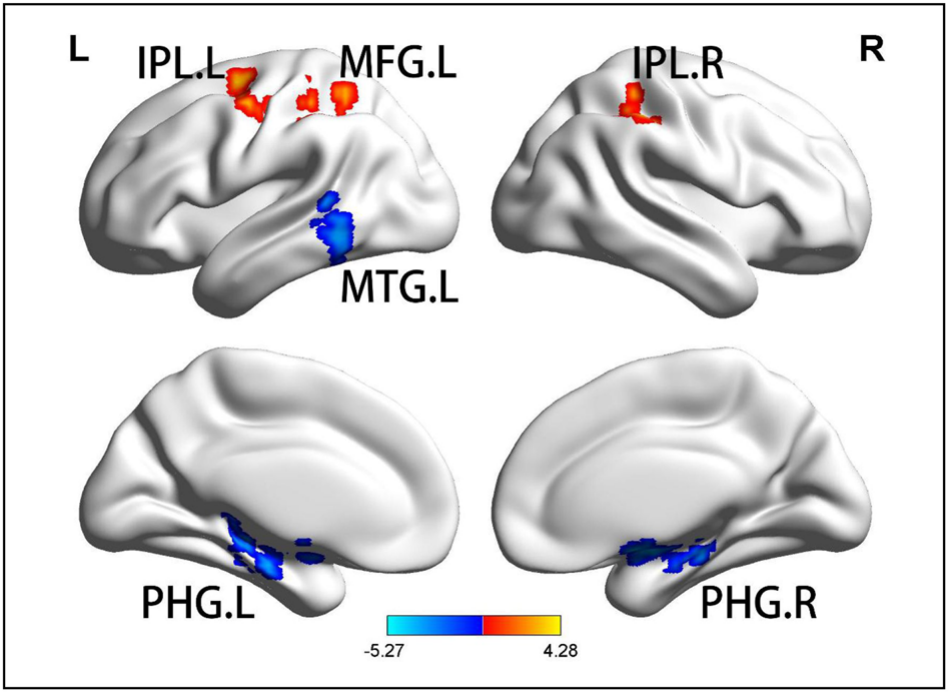
**Functional connectivity (FC) of right anterior insula (rAI) in different brain regions in the amnestic mild cognitive impairment (aMCI) and healthy control (HC) groups**. All results are displayed after adjusting for age, sex, and education. A threshold of *p* < .05 was applied, with a Gaussian random field (GRF) correction with cluster size >50 mm^3^. IPL, inferior parietal lobule; L, left; MFG, middle frontal gyrus; MTG, middle temporal gyrus; PHG, parahippocampal gyrus; R, right.

**FIGURE 3:**
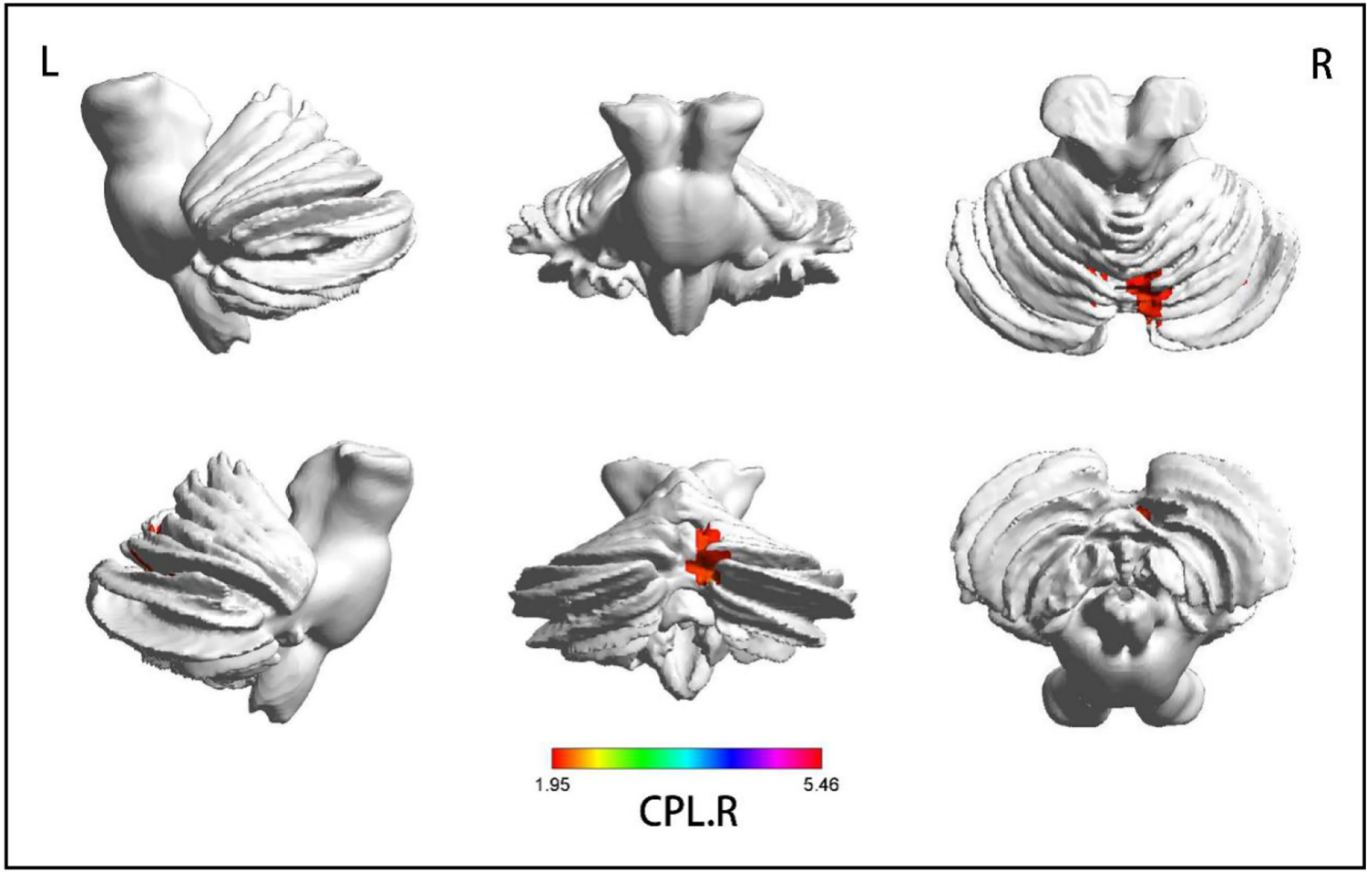
**Functional connectivity (FC) of right anterior insula (rAI) in different brain regions in the before repetitive transcranial magnetic stimulation (rTMS) and after rTMS groups**. All results are displayed after adjusting for age, sex, and education. A threshold of *p* < .05 was applied, with a Gaussian random field (GRF) correction with cluster size >50 mm^3^. ; CPL, cerebellum posterior Lobe; L, left; R, right.

Compared to HCs, subjects with aMCI had decreased FC between rAI and the left middle temporal gyrus (MTG) (*F* = −3.7501), between rAI and the bilateral parahippocampal gyrus (PHG) (*F* = −3.7038/−5.2689), and increased FC between the rAI and bilateral inferior parietal lobule (IPL) (*F* = 3.5854/4.275), and between the rAI and the left middle frontal gyrus (MFG) (*F* = 4.2807). Compared with before rTMS, only the posterior cerebellar lobes (CPL) (*F* = 5.4583) showed increased FC after rTMS (*p* < .05, GRF corrected, cluster size >50 mm^3^; Figures 2 and 3 and Table 2).

**TABLE 2:** Comparisons of functional connectivity (FC) of right anterior insula (rAI).

Brain regions	L/R	MNI			*t*‐Values	Cluster size (mm^3^)
		*X*	*Y*	*Z*		
**rAI FC**						
**aMCI vs. HC**						
Parahippocampal gyrus	L	−9	−12	−24	−3.7038	95
Parahippocampal gyrus	R	18	−6	−18	−5.2689	162
Middle temporal gyrus	L	−51	−48	−6	−3.7501	97
Inferior parietal lobule	L	−36	−36	51	3.5854	116
Inferior parietal lobule	R	48	−36	42	4.275	95
Middle frontal gyrus	L	−21	−21	54	4.2807	121
**After TMS vs. before TMS**						
Cerebellum posterior lobe	R	6	−57	−24	5.4583	354

*Note*: The *x*, *y*, *z* coordinates are the primary peak locations in the MNI space. All results are displayed after adjusting for age, sex, and education at a threshold of *p* < .05, after applying GRF correction with cluster size >50 mm^3^.

Abbreviations: aMCI, amnestic mild cognitive impairment; GRF, Gaussian random field; HC, healthy controls; L, left hemisphere; MNI, Montreal Neurological Institute; R, right hemisphere; TMS, Transcranial magnetic stimulation.

### 3.3 Correlation analysis with neuropsychological scores

**FIGURE 4:**
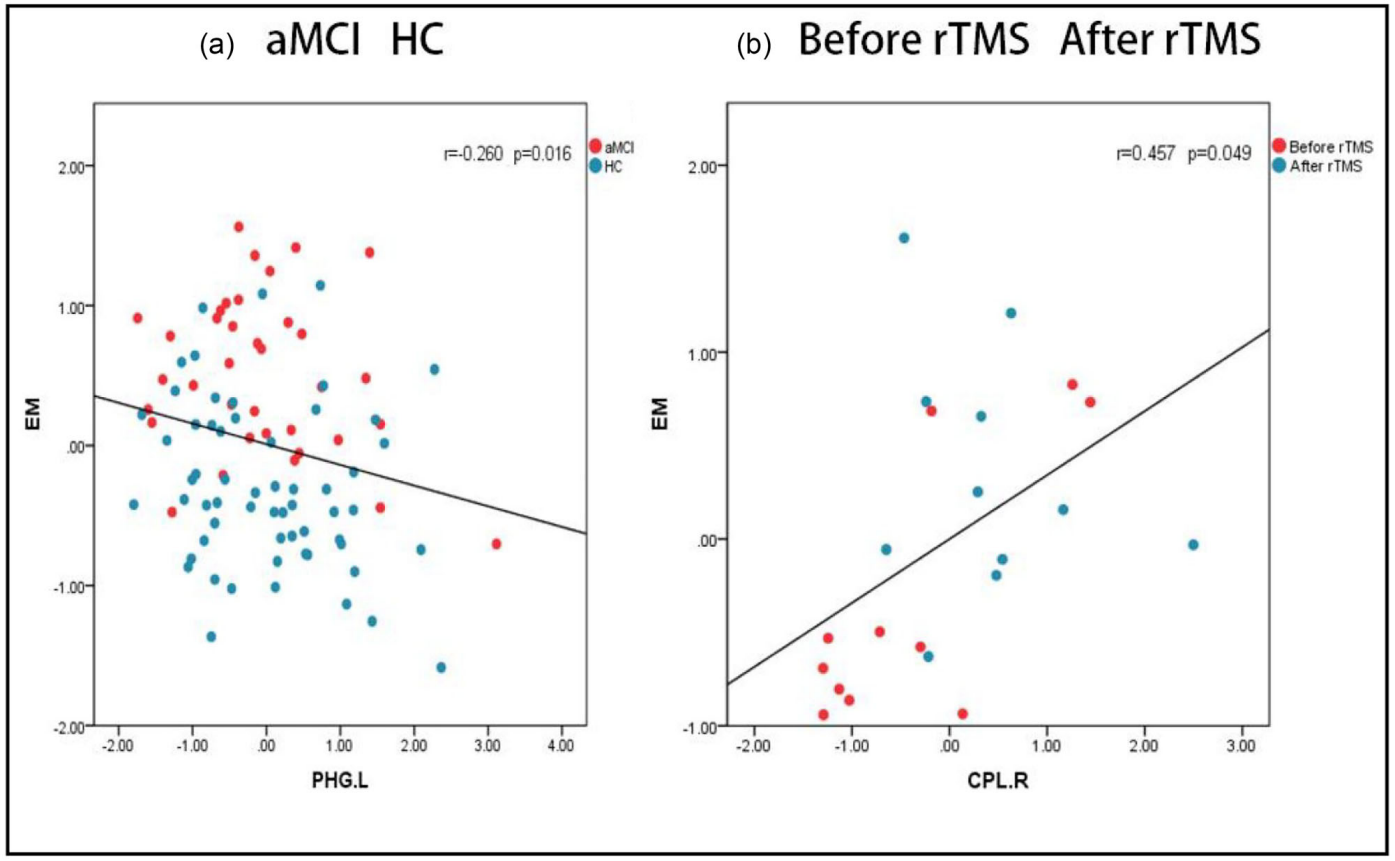
**Results of the correlation analysis: (a)** showing significant negative correlation with episodic memory (EM) score against functional connectivity (FC) between the right anterior insula (rAI) and the parahippocampal gyrus.left (PHG.L); **(b)** showing significant positive correlation with EM score against FC between the rAI and the cerebellum posterior lobe.right (CPL.R).

A correlation analysis was conducted between regions with altered FC and cognitive domains (*p* < .05). In the groups consisting of HC and aMCI, the correlation analysis showed that the altered FC between the rAI and the left PHG is negatively correlated with EM (*r* = −.260, *p* = .016). In both groups before and after rTMS, correlation analysis showed that FC changes between rAI and right CPL were positively correlated with EM (*r* = .457, *p* = .049) (Figure 4).

## 4 DISCUSSION

Our study demonstrated that targeting PCUN as a stimulatory target for rTMS not only modulates the FC of the posterior cerebellar network in patients with aMCI but also improves the cognitive status of patients with aMCI. The main finding of this study was that compared with the HC group, bilateral IPL and left MFG in aMCI patients showed an increase in FC, whereas bilateral PHG and left MTG showed a decrease in FC. At the same time, patients with aMCI after rTMS showed increased FC in the right CPL compared to those before rTMS. In addition, EM function in aMCI patients was improved after rTMS, which was closely related to FC changes in CPL. Our findings further suggest that PCUN is an effective rTMS target for aMCI progression, preventing or delaying clinical progression from aMCI to AD in therapeutic trials.

In our study, compared with the HC group, the FC between rAI and PHG was decreased in aMCI patients. The PHG, structurally part of the limbic lobe, is a diverse and complex cortex (Wotruba et al., 2014). Cognition is known to depend on intact cortical connectivity, and the hippocampus plays an important role in human memory. Interestingly, direct and indirect connections between the hippocampus and posterior cingulate cortex (PCC), which also play an important role in memory function, are made through the PHG (Soldner et al., 2012). It has been previously reported that the PHG is involved in many cognitive processes, such as EM and visuospatial processing (Aminoff et al., 2013; Burgmans et al., 2011). Meanwhile, it has also been reported that the parahippocampal posterior gyrus plays a key role in age‐related memory decline. It may be concluded that the decline of FC in PHG of aMCI patients may be closely related to the clinical manifestations of memory decline in aMCI patients. This was also consistent with our results that left PHG was negatively correlated with EM. Specifically, decreased FC between the rAI and bilateral PHG in aMCI patients may be valid biomarkers of AD transition.

It is worth mentioning that another brain region with decreased FC is the left MTG. MTG is a highly involved brain region in the language network and is thought to play a role in language‐related tasks (Briggs et al., 2021). It has been shown that functional impairment of the temporal lobe can be compensated by increased connectivity of the frontal cortical compensation network in MCI patients, thereby maintaining the cognitive status of aMCI patients, which is consistent with our results (Liang et al., 2022; Zhao et al., 2014). In our study, the left MFG showed an increase in FC. The frontal cortex is an important region for memory processing, and higher frontal cortex activity contributes to better executive memory function and EF (Liang et al., 2022; Zhao et al., 2014). This helps us to understand the relationship between FC changes and clinical manifestations in patients with aMCI and provides a theoretical basis for clinical search for effective interventions.

IPL is an important brain region of DMN, which has EM, semantic processing, and spatial cognition functions. Its changes are related to the progression from healthy aging to AD (Bzdok et al., 2016; Greene & Killiany, 2010). In our study, FC was elevated between rAI and bilateral IPL in aMCI patients, which was consistent with previous studies (Gu & Zhang, 2019; Tang et al., 2021). Previous studies have shown that the function of IPL is impaired, and FC is decreased in AD patients (Gu & Zhang, 2019; Tang et al., 2021). This functional change in hyperactivity followed by impairment may indicate a compensatory mechanism during the aMCI phase, an effort to maintain the ability to perform normal daily living. Therefore, IPL may be a biomarker of AD, and a higher FC may help maintain normal clinical manifestations of aMCI.

After rTMS, aMCI patients showed an increased FC between the rAI and right CPL. As far as we know, the cerebellum is a key part of the distributed neural circuitry that integrates and regulates the brain's network of connectivity. After cerebellar injury, the efficiency of brain network connection and information integration ability will be destroyed (Wang et al., 2019; Yao et al., 2022). The increase in FC after rTMS at PCUN is consistent with our hypothesis and may be the result of further enhancing the plasticity of cerebellar recordings and promoting associated network activity and integration in aMCI patients. At the same time, multiple cognitive scales were improved after rTMS, which may be an effective expression of rTMS in regulating cognition. Previous studies have shown that FC between the cerebellum and brain is impaired in AD patients (Wang et al., 2019; Yao et al., 2022). In the present study, increased FC was observed between the rAI and right CPL after rTMS, suggesting that PCUN may be a novel and effective target for neuromodulation in AD patients.

We targeted PCUN to study FC changes in patients with aMCI before and after rTMS. First, we looked for effective neuroimaging markers during the aMCI phase to identify early patients likely to progress to AD. Second, we found that TMS targeting PCUN can regulate brain network activity, providing new insights for clinical interventions. Finally, we further investigated the effects of TMS targeted to PCUN on cognitive function in patients with aMCI to understand the potential clinical implications of this approach.

### 4.1 Limitation

Some limitations of the current study are acknowledged. First, we had a small number of subjects, which may have reduced the credibility of the results. To avoid this possibility, we adopted GRF correction, and our database is constantly updated. Second, we did not compare the control group with false‐positive TMS stimulation, and we are collecting data on false‐positive stimulation, so we can supplement relevant studies in the future. We will continue to enrich our database in the future, hoping to improve the above deficiencies.

## 5 CONCLUSIONS

Our results suggest that effective neuroimaging markers exist in the aMCI phase, and that 10 HZ rTMS targeting PCUN may be an effective strategy for delaying the progression of patients with aMCI to AD. Specifically, our results support the use of PCUN as a new intervention target to improve cognitive performance in patients with aMCI.

## AUTHOR CONTRIBUTIONS

Qianqian Yuan, Chaoyong Xiao, and Jiu Chen designed the study. Qianqian Yuan, Chen Xue, Wenzhang Qi, Xuhong Liang, Shanshan Chen, Yu Song, Huimin Wu, Chaoyong Xiao, and Jiu Chen collected the data. Qianqian Yuan analyzed the data and prepared the manuscript. All authors contributed to the article and approved the submitted version.

## CONFLICT OF INTEREST STATEMENT

There is no conflict of interest in this article.

### PEER REVIEW

The peer review history for this article is available at https://publons.com/publon/10.1002/brb3.3169

## Supporting information

Supporting Information

## Data Availability

The original data set is available with permission by contacting our corresponding author.
